# Looking for a Signal in the Noise: Revisiting Obesity and the Microbiome

**DOI:** 10.1128/mBio.01018-16

**Published:** 2016-08-23

**Authors:** Marc A. Sze, Patrick D. Schloss

**Affiliations:** Department of Microbiology and Immunology, University of Michigan, Ann Arbor, Michigan, USA

## Abstract

Two recent studies have reanalyzed previously published data and found that when data sets were analyzed independently, there was limited support for the widely accepted hypothesis that changes in the microbiome are associated with obesity. This hypothesis was reconsidered by increasing the number of data sets and pooling the results across the individual data sets. The preferred reporting items for systematic reviews and meta-analyses guidelines were used to identify 10 studies for an updated and more synthetic analysis. Alpha diversity metrics and the relative risk of obesity based on those metrics were used to identify a limited number of significant associations with obesity; however, when the results of the studies were pooled by using a random-effect model, significant associations were observed among Shannon diversity, the number of observed operational taxonomic units, Shannon evenness, and obesity status. They were not observed for the ratio of *Bacteroidetes* and *Firmicutes* or their individual relative abundances. Although these tests yielded small *P* values, the difference between the Shannon diversity indices of nonobese and obese individuals was 2.07%. A power analysis demonstrated that only one of the studies had sufficient power to detect a 5% difference in diversity. When random forest machine learning models were trained on one data set and then tested by using the other nine data sets, the median accuracy varied between 33.01 and 64.77% (median, 56.68%). Although there was support for a relationship between the microbial communities found in human feces and obesity status, this association was relatively weak and its detection is confounded by large interpersonal variation and insufficient sample sizes.

## IMPORTANCE

As interest in the human microbiome grows, there is an increasing number of studies that can be used to test numerous hypotheses across human populations. The hypothesis that variation in the gut microbiota can explain or be used to predict obesity status has received considerable attention and is frequently mentioned as an example of the role of the microbiome in human health. Here we assessed this hypothesis by using 10 independent studies and found that although there is an association, it is smaller than can be detected by most microbiome studies. Furthermore, we directly tested the ability to predict obesity status on the basis of the composition of an individual’s microbiome and found that the median classification accuracy is between 33.01 and 64.77%. This type of analysis can be used to design future studies and expanded to explore other hypotheses.

## INTRODUCTION

Obesity is a growing health concern, with approximately 20% of the youth (aged 2 to 19) in the United States classified as either overweight or obese (1). This value increases to approximately 35% in adults (aged 20 or older), and these statistics have seen little change since 2003 (1). Traditionally, the body mass index (BMI) has been used to classify individuals as nonobese or obese (2). Recently, there has been increased interest in the role of the microbiome in modulating obesity (3, 4). If the microbiome does affect obesity status, then manipulating the microbiome could have a significant role in the future treatment of obesity and in helping to stem the current epidemic.

There have been several studies that reported observing a link between the composition of the microbiome and obesity in animal models and in humans. The first such study used genetically obese mice and observed that the ratio of the relative abundance of *Bacteroidetes* to that of *Firmicutes* (B/F ratio) was lower in obese mice than in lean mice (5). Translation of this result to humans by the same researchers did not observe this effect but did find that obese individuals had a lower alpha diversity than lean individuals (6). They also showed that the relative abundances of *Bacteroidetes* and *Firmicutes* increased and decreased, respectively, as obese individuals lost weight while on a fat- or carbohydrate-restricted diet (7). Two reanalysis studies by Walters et al. (8) and Finucane et al. (9) interrogated previously published microbiome and obesity data and concluded that the previously reported differences in community diversity and B/F ratio among nonobese and obese individuals could not be generalized. Regardless of the results obtained with human populations, studies using animal models where the community was manipulated with antibiotics or established by colonizing germfree animals with varied communities appear to support the association, since these manipulations yielded differences in animal weight (10–13). The purported association between the differences in the microbiome and obesity have been widely repeated, with little attention given to the lack of a clear signal in human cohort studies.

The recent publication of additional studies that collected BMI data for each subject, as well as other studies that were not included in the earlier reanalysis studies, offered the opportunity to revisit the question relating the structure of the human microbiome to obesity. One criticism of the prior reanalysis studies is that the authors did not aggregate the results across studies to increase the effective sample size. It is possible that there were small associations within each study that were not statistically significant because the individual studies lacked sufficient power. Alternatively, diversity metrics may mask the appropriate signal and it is necessary to measure the association at the level of microbial populations. The reanalysis study of Walters et al. demonstrated that random forest machine learning models were capable of predicting obesity status within a single cohort but did not attempt to test the models on other cohorts. The purpose of this study was to perform a meta-analysis of the association between differences in the microbiome and obesity status by analyzing and applying a more systematic and synthetic approach than was used previously.

## RESULTS

### Literature review and study inclusion.

**FIG 1:**
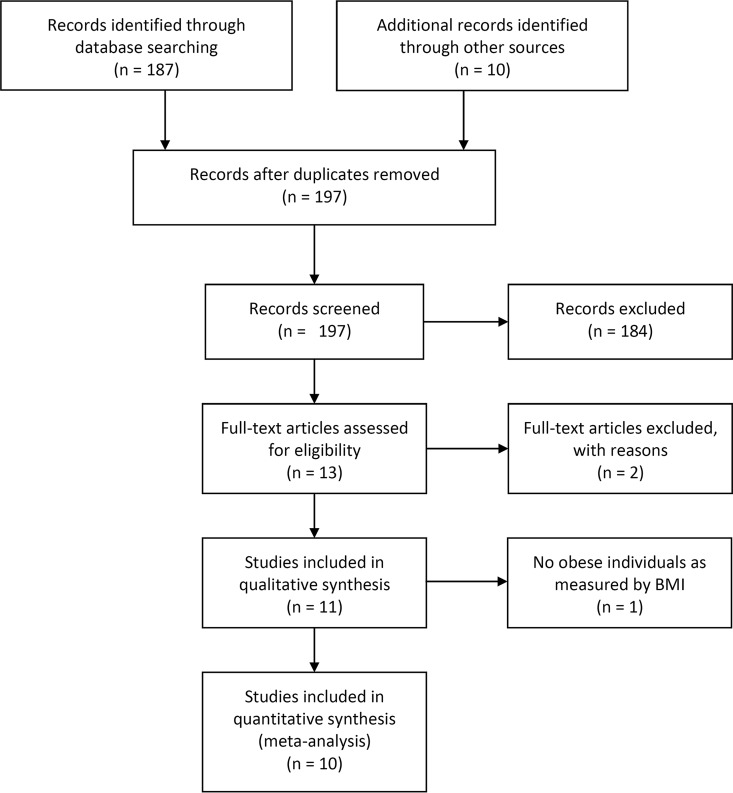
PRISMA flow diagram of all of the records searched (39).

To perform a robust meta-analysis and limit inclusion bias, we followed the preferred reporting items for systematic reviews and meta-analyses (PRISMA) guidelines to identify the studies that we analyzed (14). A detailed description of our selection process and the exact search terms are provided in Materials and Methods and Fig. 1. Briefly, we searched PubMed for original research studies that involved studying obesity and the human microbiome. The initial search yielded 187 studies. We identified 10 additional studies that were not designed to explicitly test for an association between the microbiome and obesity. We then manually curated the 197 studies to select those that included BMI and 16S rRNA gene sequence data. This yielded 11 eligible studies. An additional study was removed from our analysis because no individuals in the study had a BMI of >30. Among the final 10 studies, 3 were identified by our PubMed search (10, 15, 16), 5 were originally identified from the 10 studies that did not explicitly investigate obesity but included BMI data (17–21), and two data sets were used (22, 23) because these publications did not specifically look for any metabolic or obesity conditions but had control populations and enabled us to help mitigate against publication biases associated with the bacterial microbiome and obesity. The 10 studies are summarized in Table 1. For comparison, two of these studies were included in the reanalysis study of Finucane et al. (10, 21) and four of these studies were included in the reanalysis study of Walters et al. (10, 15, 20, 21). The 16S rRNA gene sequence data from each study were reanalyzed by a similar approach based on previously described methods for reducing the number of chimeric sequences and sequencing errors for 454 and Illumina MiSeq data (24, 25). The sequences were clustered into operational taxonomic units (OTUs) by the average-neighbor approach (26) and into taxonomic groupings based on their classification by a naive Bayesian classifier (27).

**TABLE 1:** Summary of obesity, demographic, sequencing, and beta diversity analysis data for the studies used in the meta-analysis

Study (reference)	No. of subjects	% Obese	Avg BMI (range)	% Female	Avg age in yr (range)	% Nonhispanic white	Sequencing method	16S rRNA gene region(s)	AMOVA *P* value
Baxter (23)	172	27.3	27.0 (17.5–46.9)	64.5	54.3 (29.0–80.0)	87.8	MiSeq	V4	0.078
Escobar (16)	30	33.3	27.4 (19.5–37.6)	46.7	38.1 (21.0–60.0)	NA^a^	454	V2	0.047
Goodrich (19)	982	19.7	26.3 (16.2–52.4)	98.9	61.0 (23.0–86.0)	NA	MiSeq	V4	<0.001
HMP (21)	287	10.8	24.3 (19.0–34.0)	49.1	26.3 (18.0–40.0)	81.5	454	V3–V5	0.322
Ross (18)	63	60.3	31.6 (22.1–47.9)	76.2	57.0 (33.0–81.0)	0.0	454	V1–V3	0.845
Schubert (22)	104	32.7	28.2 (18.5––62.5)	66.3	52.8 (19.0–88.0)	82.7	454	V3–V5	0.180
Turnbaugh (10)	146	67.8	NA	NA	NA	51.4	454	V2	0.040
Wu (20)	64	7.8	24.3 (14.0–41.3)	53.1	26.3 (2.16–50.0)	NA	454	V1, V2	0.577
Zeevi (17)	731	21.6	26.4 (16.4–47.0)	NA	43.4 (18.0–70.0)	NA	MiSeq	V3, V4	0.135
Zupancic (15)	207	36.2	28.2 (18.2–127.0)	57.0	46.7 (20.0–79.0)	100.0	454	V3–V5	0.206

a NA indicates that those metadata were not available for that study.

### Alpha diversity analysis.

**FIG 2:**
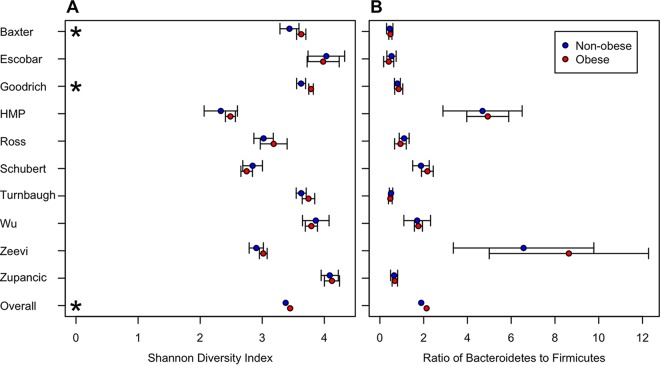
Individual and combined comparisons of obese and nonobese groups for Shannon diversity (A) and B/F ratio (B). HMP, Human Microbiome Project.

We calculated the Shannon diversity index, observed richness, and Shannon evenness, the relative abundances of *Bacteroidetes* and *Firmicutes*, and the ratio of their relative abundances (B/F ratio) for each sample. Once we transformed all six alpha diversity metrics to make them normally distributed, we used a *t* test to identify significant associations between the alpha diversity metric and whether an individual was obese for each of the 10 studies. The B/F ratio and the relative abundance of *Firmicutes* were not significantly associated with obesity in any study. We identified seven *P* values that were <0.05; three studies indicated that obese individuals had a lower richness, two studies indicated a significantly lower diversity, one study indicated a significantly lower evenness, and one study indicated a significantly higher relative abundance of *Bacteroidetes* (Fig. 2; see Fig. S1 in the supplemental material). These results largely match those of the reanalysis studies of Walters et al. and Finucane et al. Interestingly, although only 2 of the 10 studies observed the previously reported association between lower diversity and obesity, the other studies appeared to have the same trend, albeit the differences were not statistically significant. We used a random-effects linear model to combine the studies by using the study as the random effect and found statistical support for decreased richness, evenness, and diversity among obese individuals (all *P* < 0.011). Although there was a significant relationship between these metrics and obesity status, the effect size was quite small. The obese individuals averaged 7.47% lower richness, 0.88% lower evenness, and 2.07% lower diversity. There were no significant associations when we pooled the phylum-level metrics across studies. These results indicate that obese individuals do have statistically significantly lower diversity than nonobese individuals; however, it is questionable whether the difference is biologically significant.

### Relative risk.

**FIG 3:**
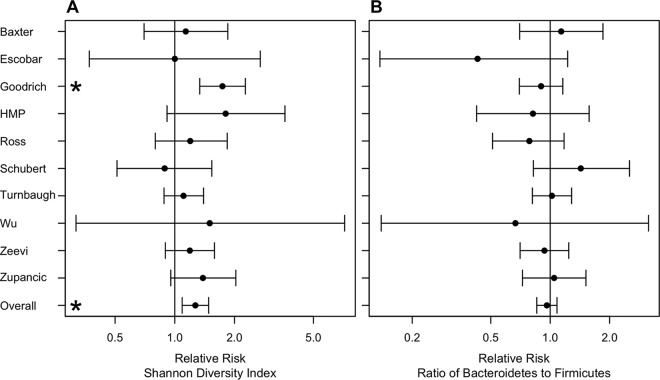
Meta-analysis of the RR of obesity based on Shannon diversity (A) or B/F ratio (B). HMP, Human Microbiome Project.

Building upon the alpha diversity analysis, we calculated the relative risk (RR) of being obese based on an individual’s alpha diversity metrics relative to the median metric for that study. Inspection of funnel plots for each of the metrics suggested that the studies included in our analysis were not biased (see Fig. S2 in the supplemental material). The results obtained by using RR largely matched those obtained by using the raw alpha diversity data. Across the 10 studies and six metrics, the only significant RR values were the richness, evenness, and diversity values from the study of Goodrich et al. (Fig. 3; see Fig. S3 in the supplemental material). Again, although the RR values were not significant for other studies, the values tended to be >1. When we pooled the data by using a random-effects model, the RR associated with having a richness, evenness, or diversity below the median for the population was significantly associated with obesity (all *P* < 0.0044). The RRs associated with alpha diversity were small. The RR of having low richness was 1.30 (95% confidence interval [CI], 1.13 to 1.49), that of having low evenness was 1.20 (95% CI, 1.06 to 1.37), and that of having low diversity was 1.27 (95% CI, 1.09 to 1.48). There were no significant differences in the phylum-level metrics. Again, the RR results indicate that although individuals with a lower richness, evenness, or diversity are at statistically significantly increased risk of being obese, it is questionable whether that risk is biologically or clinically relevant.

### Beta diversity analysis.

Following the approach used by the reanalysis studies of Walters et al. and Finucane et al., for each data set, we calculated a Bray-Curtis distance matrix to measure the difference in the membership and structure of the individuals in each study. We then used analysis of molecular variance (AMOVA) to test for significant differences between the structures of nonobese and obese individuals (Table 1). The data sets of Escobar et al., Goodrich et al., and Turnbaugh et al. indicated a significant difference in community structure (all *P* < 0.05). Because it was not possible to ascertain the directionality of the difference in community structure because the samples are arrayed in a nondimensional space or perform a pooled analysis using studies that had nonoverlapping 16S rRNA gene sequence regions, it is unclear whether these differences reflect a broader, but perhaps small, shift in community structure between nonobese and obese individuals.

### Development of a microbiome-based classifier of obesity.

**FIG 4:**
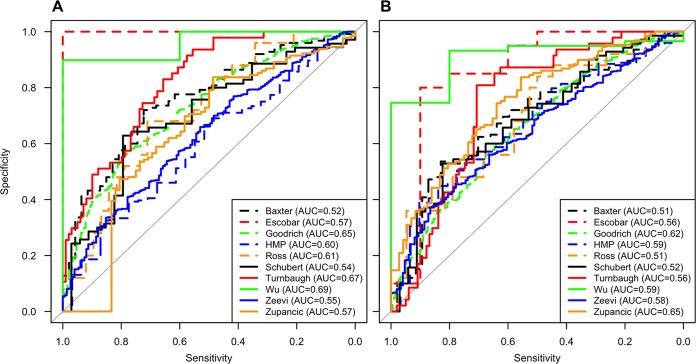
ROC curves for all of the studies based on the classification of nonobese or obese groups by using OTUs (A) or genus-level classification (B). HMP, Human Microbiome Project.

**FIG 5:**
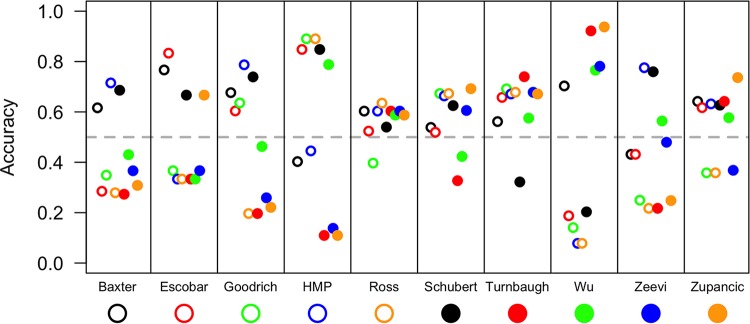
Overall accuracy with which each study predicted nonobese and obese individuals on the basis of that study’s random forest machine learning model applied to each of the other studies. HMP, Human Microbiome Project.

The reanalysis study of Walters et al. suggested that it was possible to classify individuals as being nonobese or obese on the basis of the composition of their microbiota. We repeated this analysis with additional data sets using OTU and genus-level phylotype data. For each study, we developed a random forest machine learning model to classify individuals. Using 10-fold cross validation, the cross-validated area under the curve (AUC) values for the OTU-based models varied between 0.52 and 0.69, indicating a relatively poor ability to classify individuals (Fig. 4A). To test models on other data sets, we trained models using genus-level phylotype data for each data set. The cross-validated AUC values for the models applied to the training data sets varied between 0.51 and 0.65, again indicating a relatively poor ability to classify individuals from the original data set (Fig. 4B). For each model, we identified the probability where the sum of the sensitivity and specificity was the highest. We then used this probability to define a threshold for calculating the accuracy of the models when applied to the other nine data sets (Fig. 5). Although there was considerable variation in the accuracy values for each model, the median accuracy of each model varied between 0.33 (Turnbaugh et al.) and 0.65 (Human Microbiome Project) (median, 0.57). We built similar models by using taxonomic representation based on phylum, class, order, and family assignments and saw no improvement in the results (see Fig. S4 in the supplemental material). We also attempted to predict individual BMI values as continuous variables based on the relative abundances of OTUs and genera. The median percentage of the variance explained by the resulting models was 12.9% for the OTU-based models and 8.2% for the genus-based models. When we considered the number of samples, balance of nonobese and obese individuals, and region within the 16S rRNA gene for each study, it was not possible to identify factors that predictably affected model performance. The ability to predict obesity status using relative abundance data from the communities was only marginally better than random. These results suggest that given the large diversity of microbiome compositions, it is difficult to identify a taxonomic signal that can be associated with obesity.

### Power and sample size estimate simulations.

**FIG 6:**
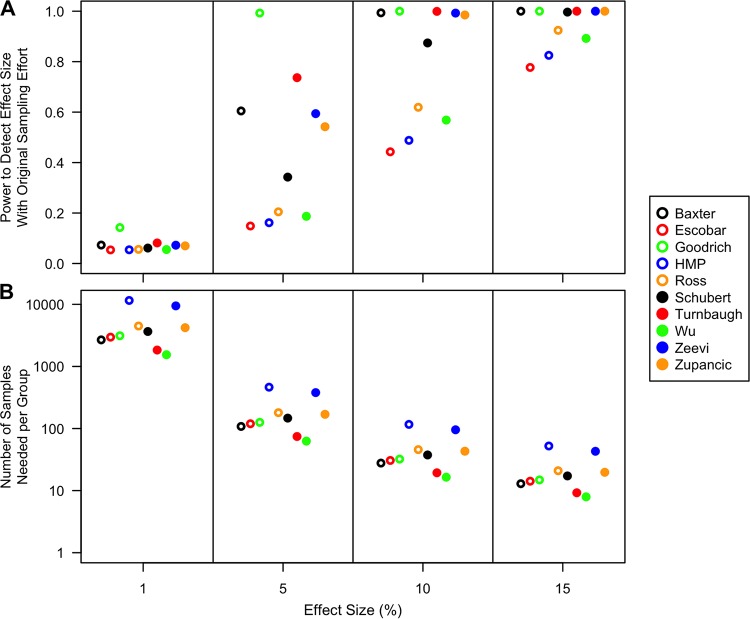
Power (A) and sample size (B) simulations for Shannon diversity for differentiating between nonobese and obese for effect sizes of 1, 5, 10, and 15%. Power calculations use the sampling distribution from the original studies, and the sample size estimations assume an equal amount of sampling from each treatment group. HMP, Human Microbiome Project.

The inability to detect a difference between nonobese and obese individuals could be due to the lack of a true effect or because the study had insufficient statistical power to detect a difference because of insufficient sampling, large interpersonal variation, or unbalanced sampling of nonobese and obese individuals. To assess these factors, we calculated the power to detect differences of 1, 5, 10, and 15% in each of the alpha diversity metrics by using the sample sizes used in each of the studies (Fig. 6; see Fig. S5 to S10 in the supplemental material). Although there is no biological rationale for these effect sizes, they represent a range that includes effect sizes that would be generally considered biologically significant. Only the study of Goodrich et al. had a power of >0.80 to detect a 5% difference in the Shannon diversity, and six of the studies had enough power to detect a 10% difference (Fig. 6A). None of the studies had sufficient power to detect a 15% difference between B/F ratios (see Fig. S5 in the supplemental material). In fact, the maximum power among any of the studies to detect a 15% difference in B/F ratios was 0.25. Among the tests for RR, none of the studies had sufficient power to detect a Cohen *d* value of 0.10 and only two studies had sufficient power to detect a Cohen *d* value of 0.15. We next estimated how many individuals would need to be sampled to have sufficient power to detect the four effect sizes assuming the observed interpersonal variation from each study and balanced sampling between the two groups (Fig. 6B). To detect a 1, 5, 10, or 15% difference in Shannon diversity, the median required sampling effort per group was approximately 3,400, 140, 35, or 16 individuals, respectively. To detect a 1, 5, 10, or 15% difference in B/F ratios, the median required sampling effort per group was approximately 160,000, 6,300, 1,600, or 700 individuals, respectively. To detect a 1, 5, 10, or 15% difference in RR values using Shannon diversity, the median required sampling effort per group was approximately 39,000, 1,500, 380, or 170 individuals, respectively. These estimates indicate that most microbiome studies lack the power to detect modest effect sizes by using either metric. In the case of obesity, the studies lack the power to detect the 0.90 to 6% difference in diversity that was observed across the studies.

## DISCUSSION

Our meta-analysis helps to provide clarity to the ongoing debate of whether or not there are specific microbiome-based markers that can be associated with obesity. We performed an extensive literature review of the existing studies on the microbiome and obesity and performed a meta-analysis of the studies that remained on the basis of our inclusion and exclusion criteria. By statistically pooling the data from 10 studies, we observed significant, but small, relationships between richness, evenness, and diversity and obesity status, as well as the RR of being obese based on these metrics. We also generated random forest machine learning models trained on each data set and tested on the remaining data sets. This analysis demonstrated that the ability to reliably classify individuals as obese solely on the basis of the composition of their microbiome was limited. Finally, we assessed the ability of each study to detect defined differences in alpha diversity and observed that most studies lacked the power to detect modest effect sizes. Considering that these data sets are among the largest published, it appears that most human microbiome studies lack the power to detect differences in alpha diversity.

Alpha diversity metrics are attractive because they distill a complex data set to a single value. For example, Shannon diversity is a measurement of the entropy in a community and integrates richness and evenness information. Two communities with little taxonomic similarity can have the same diversity. Among ecologists, the relevance of these metrics is questioned because it is difficult to ascribe a mechanistic interpretation to their relationship with stability or disease. Regardless, the concept of a biologically significant effect size needs to be developed among microbiome researchers. Alternative metrics could include the ability to detect a defined difference in the relative abundance of an OTU representing a defined relative abundance. What makes for a biologically significant difference or relative abundance is an important point that has yet to be discussed in the microbiome field. The use of operationally defined effect sizes should be adequate until it is possible to decide upon an accepted practice.

By selecting a range of possible effect sizes, we were able to demonstrate that most studies lack the power to detect modest differences in alpha diversity metrics and phylum-level relative abundances. Several factors interact to limit the power of microbiome studies. There is wide interpersonal variation in the diversity and structure of the human microbiome. Some factors, such as the relationship between subjects, could potentially decrease the amount variation (6), and other factors, such as whether one lives in a rural environment, could increase the amount of variation (28). In addition, the most common experimental designs limit their power. As we observed, most of the studies included in our analysis were unbalanced for the variable that we were interested in. This was also true of those studies that originally sought to identify associations with obesity. Even with a balanced design, we showed that it was necessary to obtain approximately 140 and 6,300 samples per group to detect a 5% difference in Shannon diversity or B/F ratio, respectively. It was interesting that these sample sizes agreed across studies regardless of their sequencing method, region within the 16S rRNA gene, or subject population (Fig. 6). This suggests that, regardless of the treatment or category, these sample sizes represent a good starting point for subject recruitment when using stool samples. Unfortunately, few studies with this level of subject recruitment have been published. This is troubling, since the positive predictive rate of a significant finding in an underpowered study is small, leading to results that cannot be reproduced (29). Future microbiome studies should articulate the basis for their experimental design.

Two previous reanalysis studies have stated that there was not a consistent association between alpha diversity and obesity (8, 9); however, neither of these studies made an attempt to pool the existing data to try to harness the additional power that this would give, and they did not assess whether the studies were sufficiently powered to detect a difference. Additionally, our analysis used 16S rRNA gene sequence data from 10 studies, whereas the study of Finucane et al. used 16S rRNA gene sequence data from three studies (7, 10, 21) and a metagenomic study (30) and the study of Walters et al. used 16S rRNA gene sequence data from five studies (10, 15, 20, 21, 28); two studies were included in both analyses (10, 21). Our analysis included four of these studies (10, 15, 20, 21) and excluded three of the studies because they were too small (7), only utilized metagenomic data (30), or used short, single-read Illumina HiSeq data that have a high error rate, making them intractable for *de novo* OTU clustering (28). The additional seven data sets were published after the two reviews were performed and include data sets with more samples than were found in the original studies. Our collection of 10 studies allowed us to largely use the same sequence analysis pipeline for all of the data sets and relied heavily on the availability of public data and access to metadata that included variables beyond the needs of the original study. To execute this analysis, we created an automated data analysis pipeline, which can be easily updated to add additional studies as they become available (https://github.com/SchlossLab/Sze_Obesity_mBio_2016/). Similarly, it would be possible to adapt this pipeline to other body sites and treatment or variables (e.g., subject sex or age).

Similar to our study, the study of Walters et al. generated random forest machine learning models to differentiate between nonobese and obese individuals (8). They obtained AUC values similar to those of our analysis; however, they did not attempt to test these models on the other studies in their analysis. When we performed cross validation of data sets, the median accuracy across the data sets was only 56.68%, indicating that the models did a poor job when applied to other data sets. This could be due to differences in subject populations and methods. Furthermore, others have reported improved classification at broader taxonomic levels (31); we did not find this to be the case across the studies in our analysis (see Fig. S4 in the supplemental material). Considering that the median AUC for models trained and tested on the same data with 10-fold cross validation only varied between 0.51 and 0.65 and that there was not a strong signal in the alpha diversity data, we suspect that there is insufficient signal to reliably classify individuals in a BMI category on the basis of their microbiota.

Although we failed to find an effect, this does not necessarily mean that there is no role for the microbiome in obesity. There is strong evidence in murine models of obesity that the microbiome and level of adiposity can be manipulated via genetic manipulation of the animal and manipulation of the community through antibiotics or colonization of germfree mice with diverse fecal material from human donors (5, 10–13). These studies appear to conflict with the observations based on human subjects. Recalling the large interpersonal variation in the structure of the microbiome, it is possible that each individual has his or her own signatures of obesity. Alternatively, it could be that the involvement of the microbiome in obesity is not apparent based on the taxonomic information provided by 16S rRNA gene sequence data. Rather, the differences could become more apparent at the level of a common set of gene transcripts or metabolites that can be produced from different structures of the microbiome.

## MATERIALS AND METHODS

### In-depth overview of search strategy.

The initial search strategy included looking for all papers that initially fit under the following NCBI PubMed advanced search criteria: “(((((((((Bacterial Microbiome) AND (Obesity or bmi or body mass index or BMI or obesity) AND”last 10 years“[PDat] AND Humans[Mesh])) NOT review[ptyp]) AND”last 10 years“[PDat] AND Humans[Mesh])) AND”last 10 years“[PDat] AND Humans[Mesh])) AND”Last 10 years“[PDat] AND Humans[Mesh]).” The report had to have the terms “Bacterial Microbiome” and “Obesity, BMI, bmi, obesity” in its criteria, it could not have been published >10 years ago, it could not be a review article, and it had to contain research on humans only. This search yielded a total of 187 reports. From the reanalysis studies of Finucane et al. and Walters et al. along with knowledge of other published papers that included BMI information in their sequence metadata, we obtained 10 additional studies. This brought our total number of records to 197. We browsed the abstracts of these studies and included studies that mentioned stool or feces examination, did not involve children, were not clinical trials for probiotics or other diet-related treatments, did not have participants with inflammatory bowel disease, were in English, and were based on the analysis of sequence data from the 454 or MiSeq platform. This ultimately excluded all but a total of 11 studies. Because we decided *a priori* to use the standard definition for BMI group classification, one study was excluded because it did not have any individuals who were obese.

### Sequence analysis pipeline.

All sequence data were publicly available and were downloaded from the NCBI Sequence Read Archive, the European Nucleotide Archive, or the investigators’ personal website (https://gordonlab.wustl.edu/NatureTwins_2008/TurnbaughNature_11_30_08.html). Seven studies used 454 sequencing (6, 15, 16, 18, 20–22), and three used Illumina sequencing (17, 19, 23). All of these studies used amplification-based 16S rRNA gene sequencing. Among the studies that sequenced the 16S rRNA gene, the researchers targeted the V1 to V2 (20), V1 to V3 (15, 16, 18), V3 to V5 (21, 22), V4 (19, 23), and V3 to V4 (17) variable regions. For those studies where multiple regions were sequenced, we selected the region that corresponded to the largest number of subjects (6, 21). We processed the 16S rRNA gene sequence data by using a standardized mothur pipeline. Briefly, our pipelines attempted to follow previously recommended approaches for 454 and Illumina sequencing data (24, 25). All sequences were screened for chimeras with UCHIME and assigned to OTUs by using the average-neighbor algorithm with a 3% distance threshold (26, 32). All sequence processing was performed with mothur (version 1.37.0) (33).

### Data analysis.

We split the overall meta-analysis into three general strategies by using R (3.3.0). First, we used the approach employed by Finucane et al. (9) and Walters et al. (8), where each study was reanalyzed separately to identify associations between BMI and the relative abundances of *Bacteroidetes* and *Firmicutes*, the ratio of the relative abundances of *Bacteroidetes* and *Firmicutes* (B/F ratio), Shannon diversity, observed richness, and Shannon evenness (34). After each variable was transformed to fit a normal distribution, a two-tailed *t* test was performed for comparison of nonobese and obese individuals (i.e., those with a BMI of >35.0). We performed a pooled analysis of these measured variables by using linear random-effects models to correct for study effect to assess differences in the combined data set between nonobese and obese groups by using the lme4 (version 1.1-12) R package (35). Next, we compared the community structure from nonobese and obese individuals by using AMOVA with Bray-Curtis distance matrices (36). This analysis was performed with the vegan (version 2.3-5) R package. For both analyses, the data sets were rarefied (*n =* 1,000) so that each study had the same number of sequences. Second, for each study, we partitioned the subjects into a low or high group, depending on whether their alpha diversity metrics were below or above the median value for the study. The RR was then calculated as the ratio of the number of obese individuals in the low group to the number of obese individuals in the high group. We then performed a Fisher exact test to investigate whether the RR was significantly different from 1.0 within each study and across all of the studies by using the epiR (version 0.9-77) and metafor (version 1.9-8) packages. Third, we used the AUCRF (version 1.1) R package to generate random forest models (37). For each study, we developed models using either OTUs or genus-level phylotypes. The quality of each model was assessed by measuring the AUC of the receiver operating characteristic (ROC) with 10-fold cross validation. Because the genus-level phylotype models were developed with a common reference, it was possible to use one study’s model (i.e., the training set) to classify the samples from the other studies (i.e., the testing sets). The optimum threshold for the training set was set as the probability threshold that had the highest combined sensitivity and specificity. This threshold was then used to calculate the accuracy of the model applied to the test studies. To generate ROC curves and calculate the accuracy of the models, we used the pROC (version 1.8) R package (38). Finally, we performed power and sample number simulations for different effect sizes for each study using the pwr (version 1.1-3) R package and base R functions. We also calculated the actual sample size needed on the basis of the effect size of each individual study.

### Reproducible methods.

A detailed and reproducible description of how the data were processed and analyzed can be found at https://github.com/SchlossLab/Sze_Obesity_mBio_2016/.

## SUPPLEMENTAL MATERIAL

Figure S1  Individual and combined comparisons of obese and nonobese groups based on evenness (A), richness (B), or the relative abundances of *Bacteroidetes* (C) and *Firmicutes* (D). Download Figure S1, PDF file, 0.2 MB

Figure S2  Funnel plots depicting the general lack of bias in the selection of data sets included in the analysis. Download Figure S2, PDF file, 0.3 MB

Figure S3  Meta-analysis of the RR of obesity based on evenness (A), richness (B), or the relative abundances of *Bacteroidetes* (C) and *Firmicutes* (D). Download Figure S3, PDF file, 0.2 MB

Figure S4  Overall accuracy with which each study predicted nonobese and obese individuals based on that study’s random forest machine learning model applied to each of the other studies when trained by using the relative abundance of each phylum, class, order, family, or genus. The cross-validated AUC values for the training model are provided for each study and taxonomic level. Download Figure S4, PDF file, 0.5 MB

Figure S5  Power (A) and sample size (B) simulations for B/F ratio for differentiating between nonobese and obese for effect sizes of 1, 5, 10, and 15%. Power calculations use the sampling distribution from the original studies, and the sample size estimations assume the same amount of sampling from each treatment group. Download Figure S5, PDF file, 0.2 MB

Figure S6  Power (A) and sample size (B) simulations for richness for differentiating between nonobese and obese for effect sizes of 1, 5, 10, and 15%. Power calculations use the sampling distribution from the original studies, and the sample size estimations assume the same amount of sampling from each treatment group. Download Figure S6, PDF file, 0.2 MB

Figure S7  Power (A) and sample size (B) simulations for evenness for differentiating between nonobese and obese for effect sizes of 1, 5, 10, and 15%. Power calculations use the sampling distribution from the original studies, and the sample size estimations assume the same amount of sampling from each treatment group. Download Figure S7, PDF file, 0.2 MB

Figure S8  Power (A) and sample size (B) simulations for the relative abundance of *Bacteroidetes* for differentiating between nonobese and obese for effect sizes of 1, 5, 10, and 15%. Power calculations use the sampling distribution from the original studies, and the sample size estimations assume the same amount of sampling from each treatment group. Download Figure S8, PDF file, 0.2 MB

Figure S9  Power (A) and sample size (B) simulations for the relative abundance of *Firmicutes* for differentiating between nonobese and obese for effect sizes of 1, 5, 10, and 15%. Power calculations use the sampling distribution from the original studies, and the sample size estimations assume the same amount of sampling from each treatment group. Download Figure S9, PDF file, 0.2 MB

Figure S10  Power (A) and sample size (B) simulations for RR of obesity based on Shannon diversity. Power calculations use the sampling distribution from the original studies, and the sample size estimations assume the same amount of sampling from each treatment group. Download Figure S10, PDF file, 0.2 MB
