# Erratum for Sze and Schloss, “Looking for a Signal in the Noise: Revisiting Obesity and the Microbiome”

**DOI:** 10.1128/mBio.01995-17

**Published:** 2017-12-05

**Authors:** Marc A. Sze, Patrick D. Schloss

**Affiliations:** Department of Microbiology and Immunology, University of Michigan, Ann Arbor, Michigan, USA

## ERRATUM

Volume 7, no. 4, e01018-16, 2016, https://doi.org/10.1128/mBio.01018-16. The definitions of the colored dots in Fig. 2 and Fig. S1 in the supplemental material were inadvertently reversed. The text in the body of the paper correctly describes the colors in the figures (blue indicates obesity, and red indicates nonobesity). This change does not affect the conclusions of the paper.

The following funding and data availability information should have been included.

**Availability of data.** The data set is available from dbGaP as part of NIH Human Microbiome Project Core Microbiome Sampling Protocol A (HMP-A) under accession no. phs000228.v3.p1.

