# Supplementary figures and images for: Looking for a Signal in the Noise: Revisiting Obesity and the Microbiome
Source: mBio. 2016 Aug 23;7(4):e01018-16. doi: 10.1128/mBio.01018-16 (PMC4999546; doi:10.1128/mBio.01018-16)

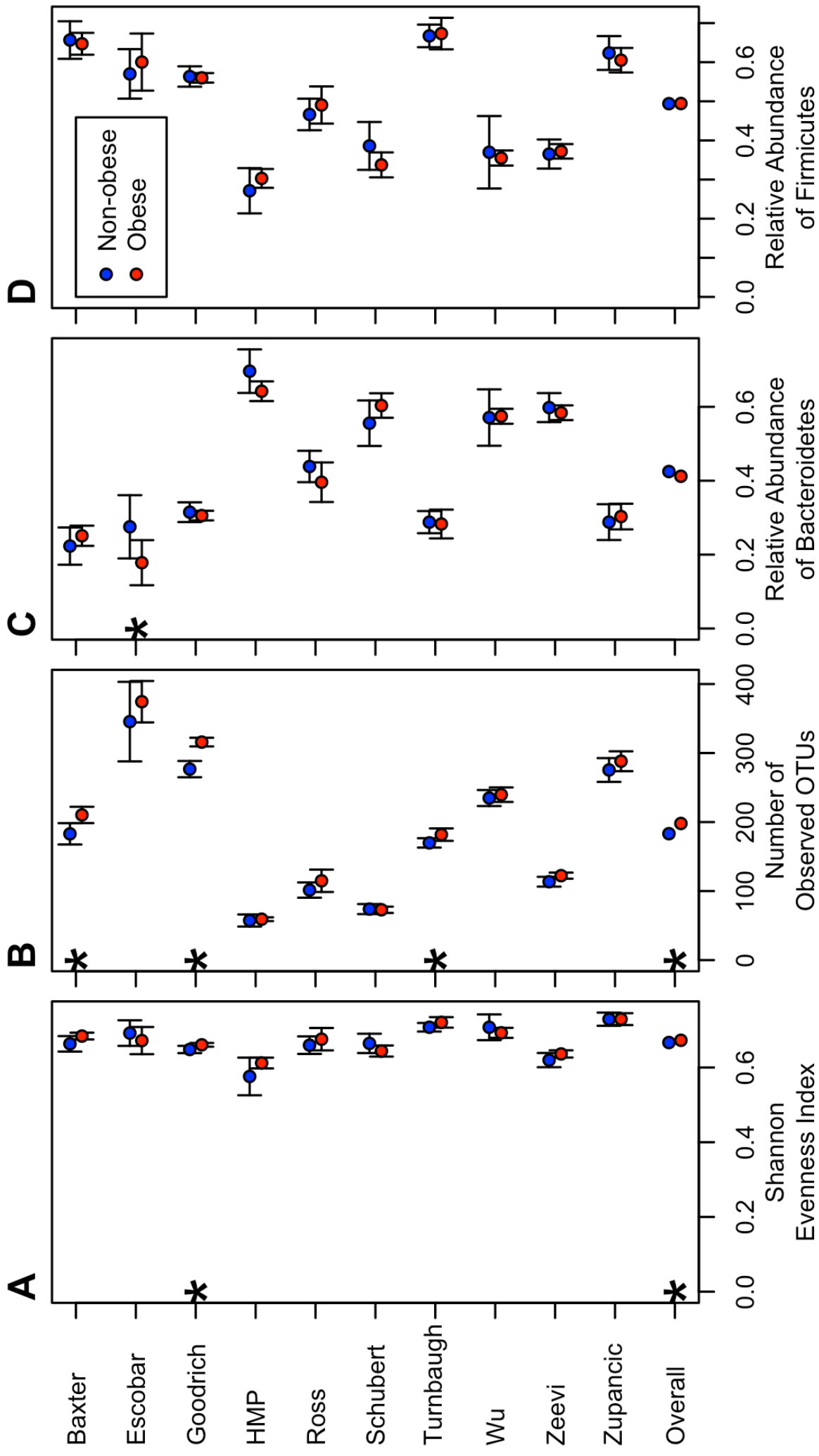

Supplement: Figure S1 — Individual and combined comparisons of obese and nonobese groups based on evenness (A), richness (B), or the relative abundances of Bacteroidetes (C) and Firmicutes (D). Download [file mbo004162954sf1.pdf]

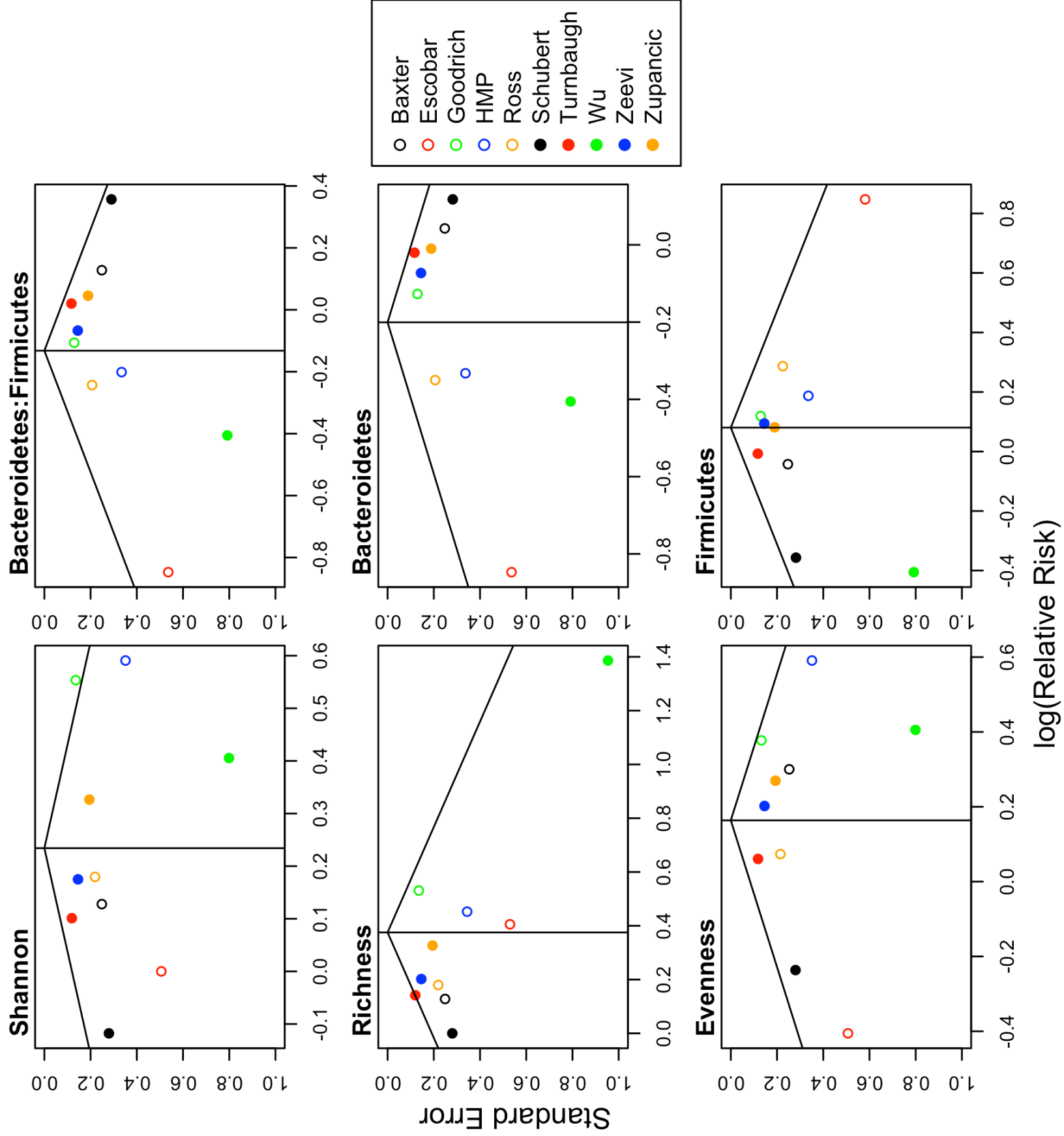

Supplement: Figure S2 — Funnel plots depicting the general lack of bias in the selection of data sets included in the analysis. Download [file mbo004162954sf2.pdf]

**A**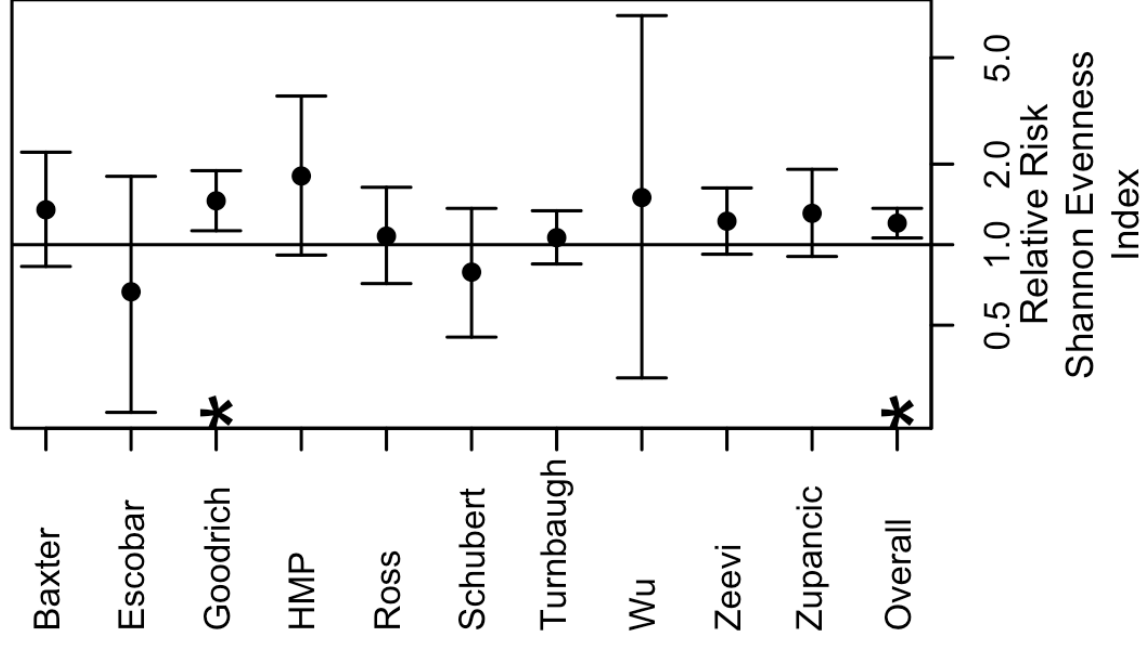**B**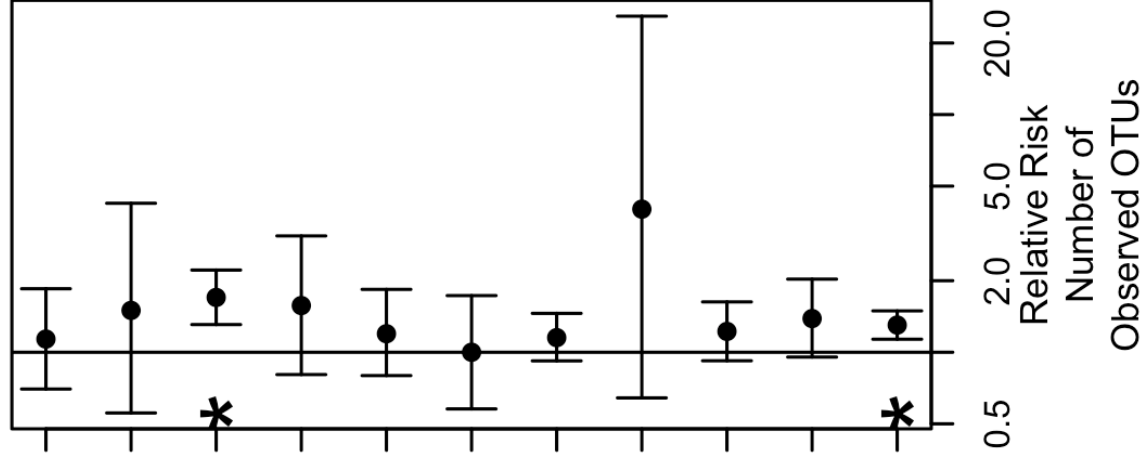**C**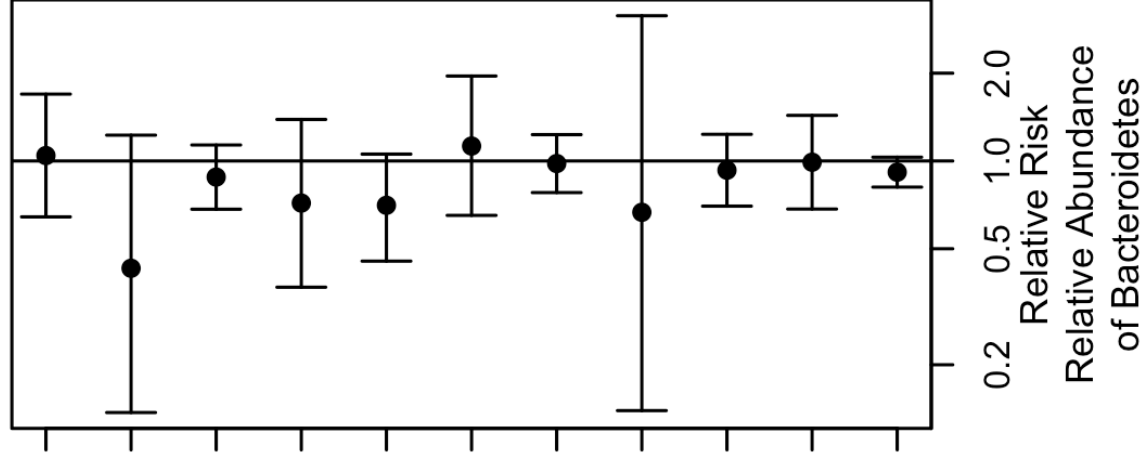**D**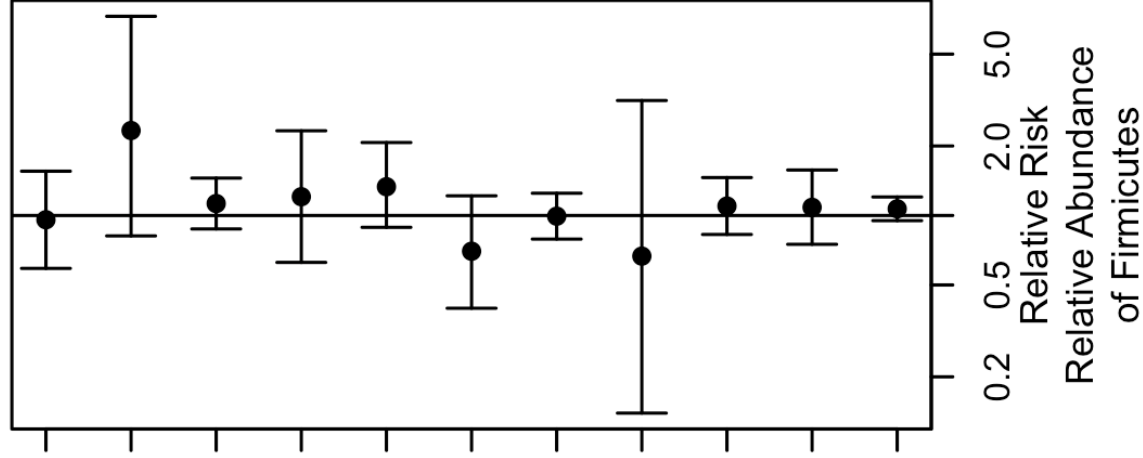

Supplement: Figure S3 — Meta-analysis of the RR of obesity based on evenness (A), richness (B), or the relative abundances of Bacteroidetes (C) and Firmicutes (D). Download [file mbo004162954sf3.pdf]

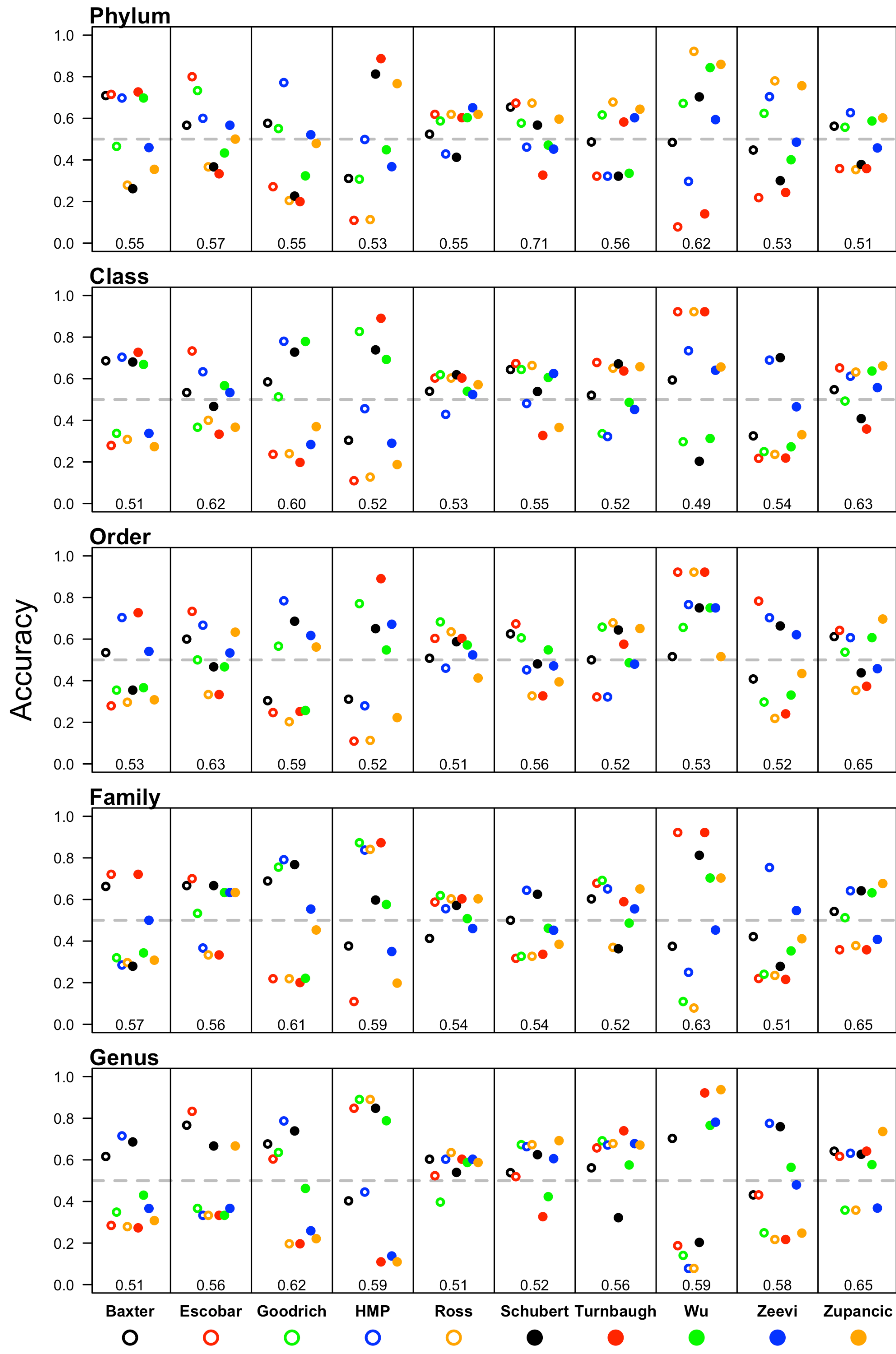

Supplement: Figure S4 — Overall accuracy with which each study predicted nonobese and obese individuals based on that study’s random forest machine learning model applied to each of the other studies when trained by using the relative abundance of each phylum, class, order, family, or genus. The cross-validated AUC values for the training model are provided for each study and taxonomic level. Download [file mbo004162954sf4.pdf]

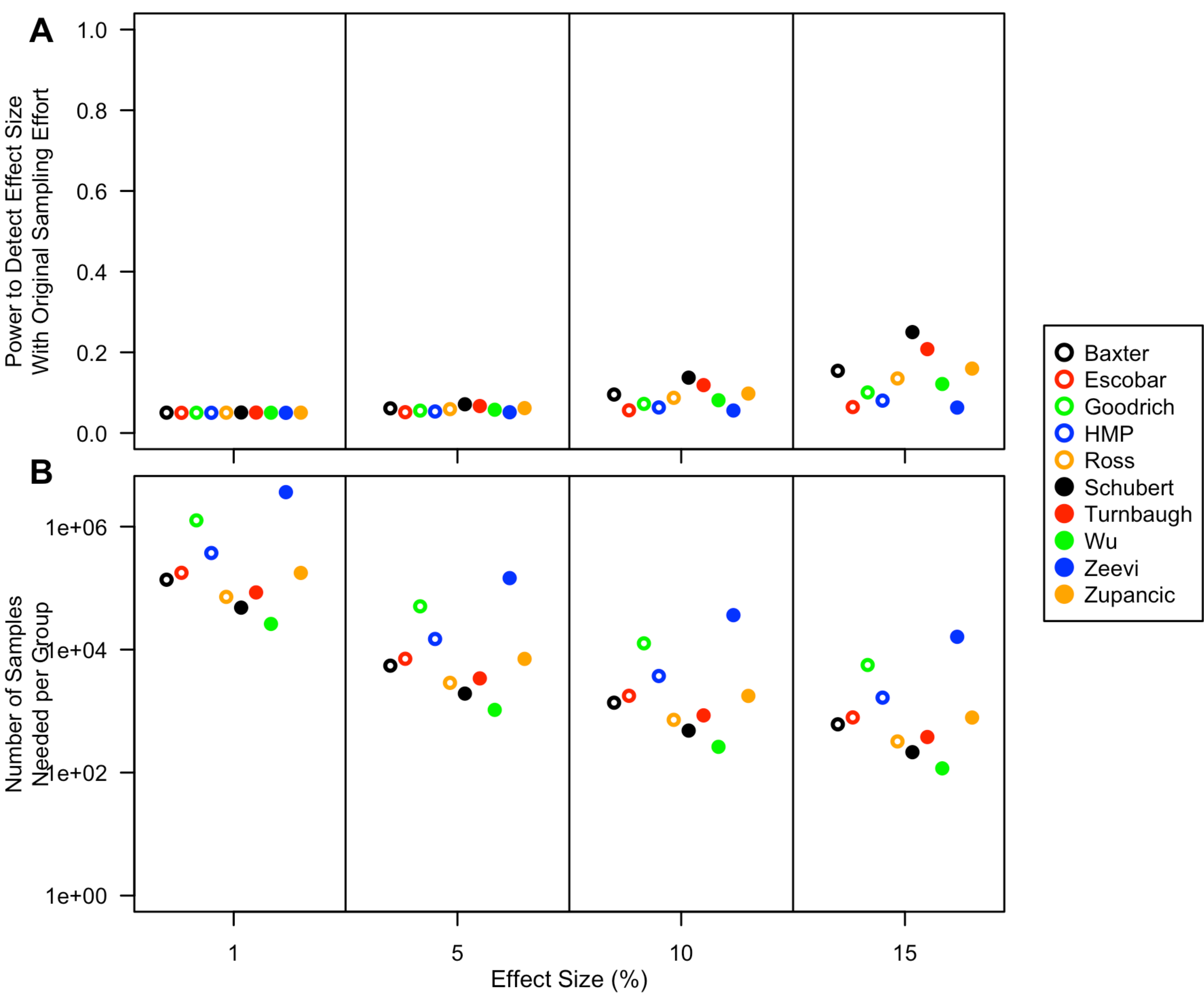

Supplement: Figure S5 — Power (A) and sample size (B) simulations for B/F ratio for differentiating between nonobese and obese for effect sizes of 1, 5, 10, and 15%. Power calculations use the sampling distribution from the original studies, and the sample size estimations assume the same amount of sampling from each treatment group. Download [file mbo004162954sf5.pdf]

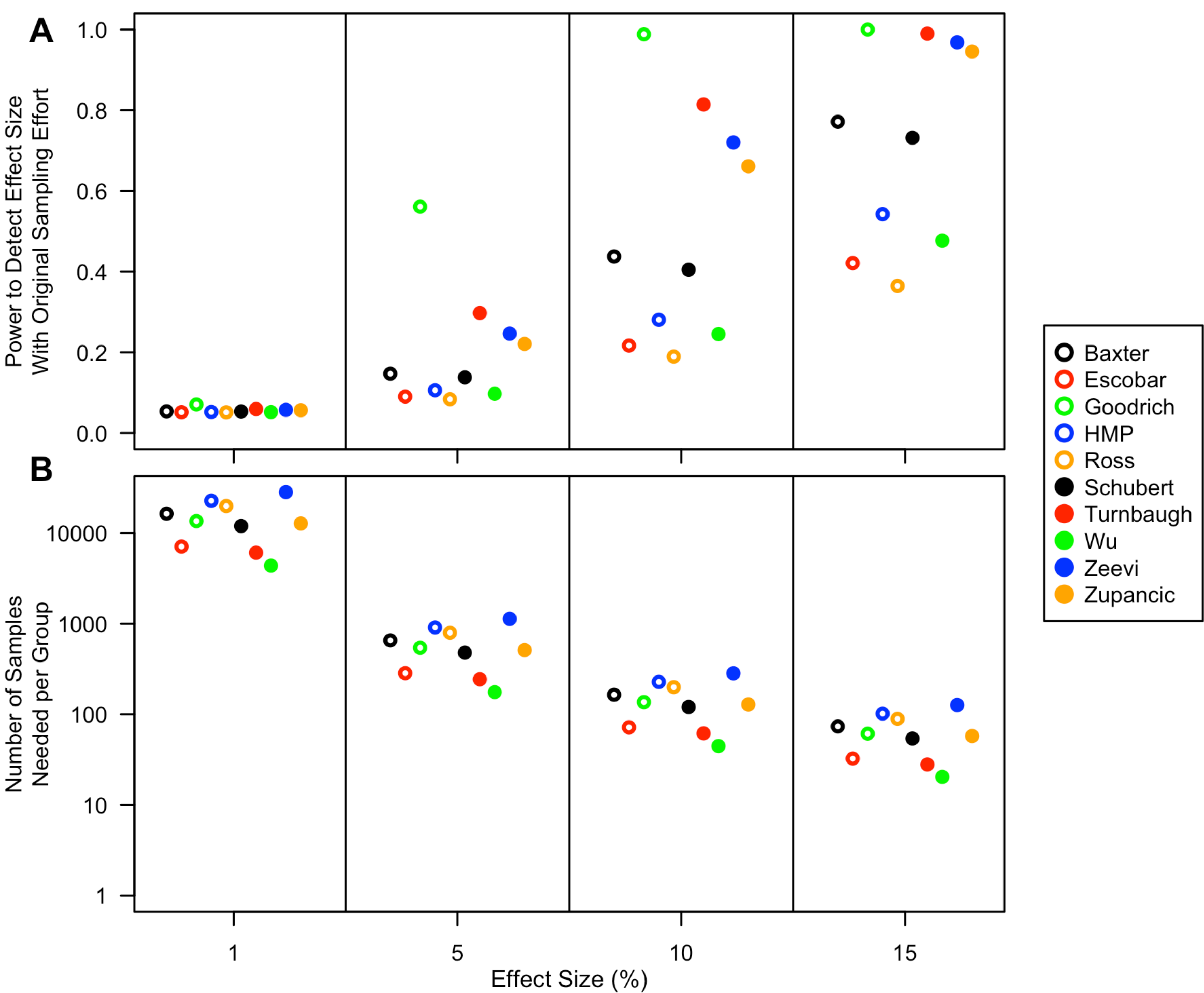

Supplement: Figure S6 — Power (A) and sample size (B) simulations for richness for differentiating between nonobese and obese for effect sizes of 1, 5, 10, and 15%. Power calculations use the sampling distribution from the original studies, and the sample size estimations assume the same amount of sampling from each treatment group. Download [file mbo004162954sf6.pdf]

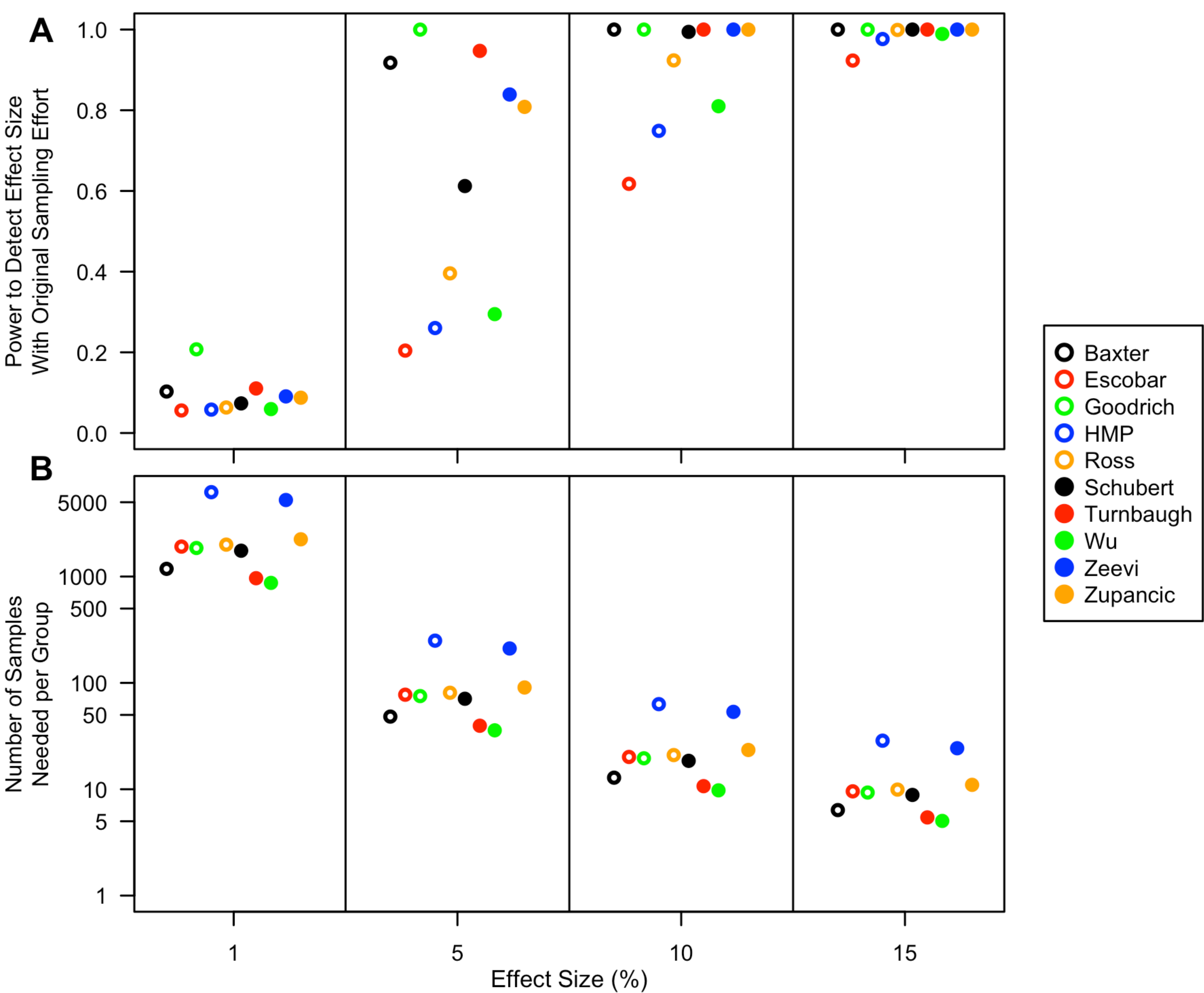

Supplement: Figure S7 — Power (A) and sample size (B) simulations for evenness for differentiating between nonobese and obese for effect sizes of 1, 5, 10, and 15%. Power calculations use the sampling distribution from the original studies, and the sample size estimations assume the same amount of sampling from each treatment group. Download [file mbo004162954sf7.pdf]

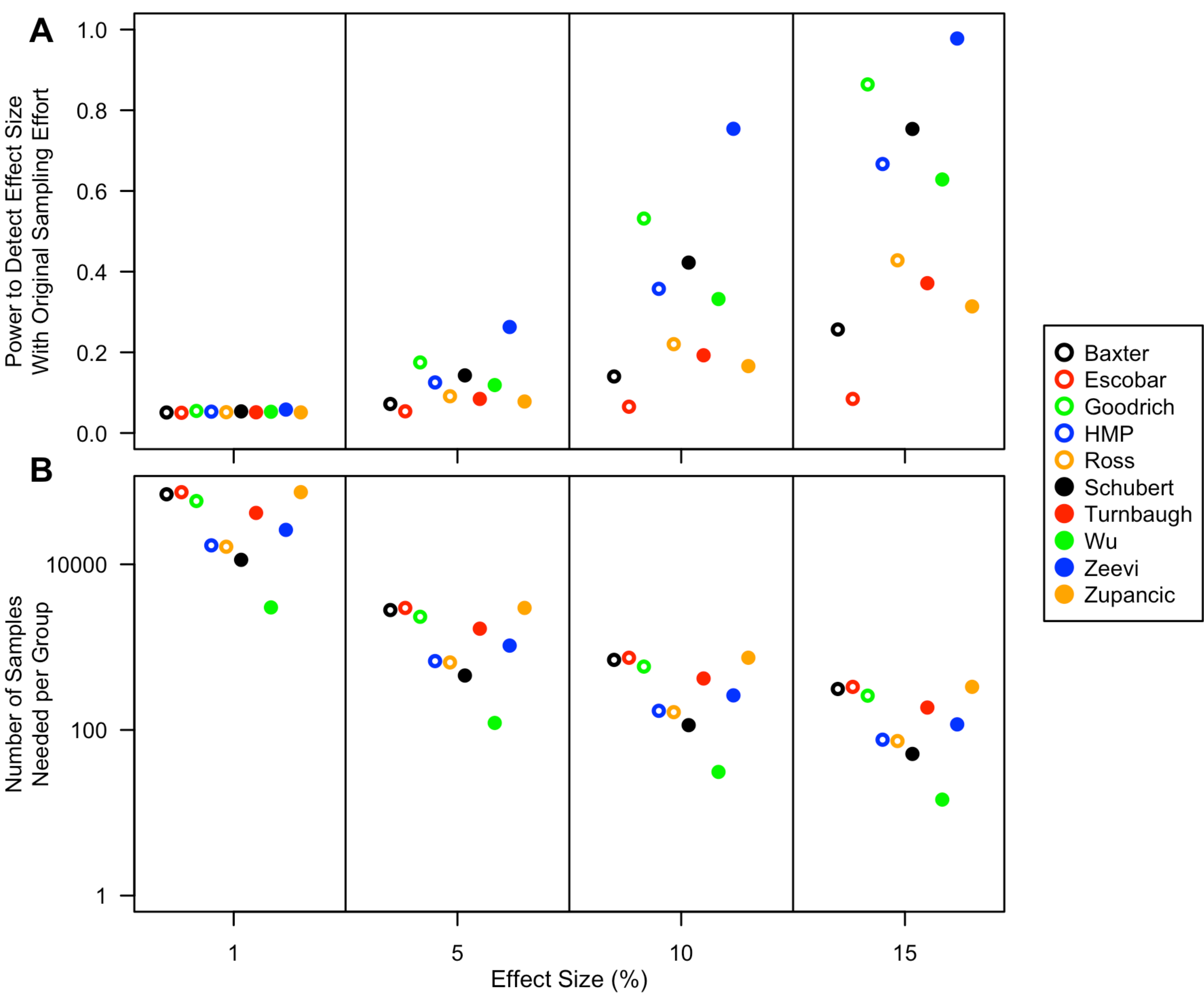

Supplement: Figure S8 — Power (A) and sample size (B) simulations for the relative abundance of Bacteroidetes for differentiating between nonobese and obese for effect sizes of 1, 5, 10, and 15%. Power calculations use the sampling distribution from the original studies, and the sample size estimations assume the same amount of sampling from each treatment group. Download [file mbo004162954sf8.pdf]

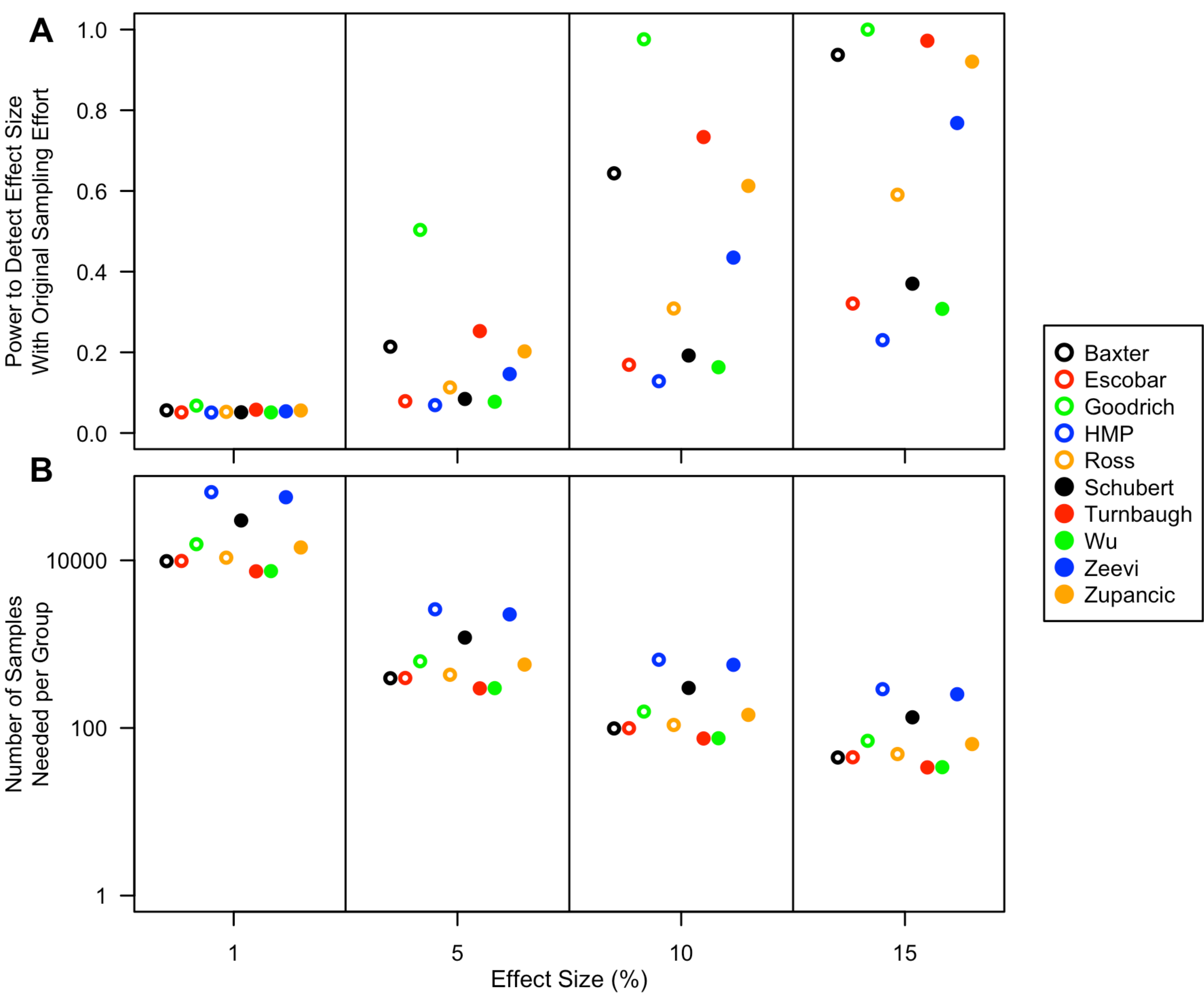

Supplement: Figure S9 — Power (A) and sample size (B) simulations for the relative abundance of Firmicutes for differentiating between nonobese and obese for effect sizes of 1, 5, 10, and 15%. Power calculations use the sampling distribution from the original studies, and the sample size estimations assume the same amount of sampling from each treatment group. Download [file mbo004162954sf9.pdf]

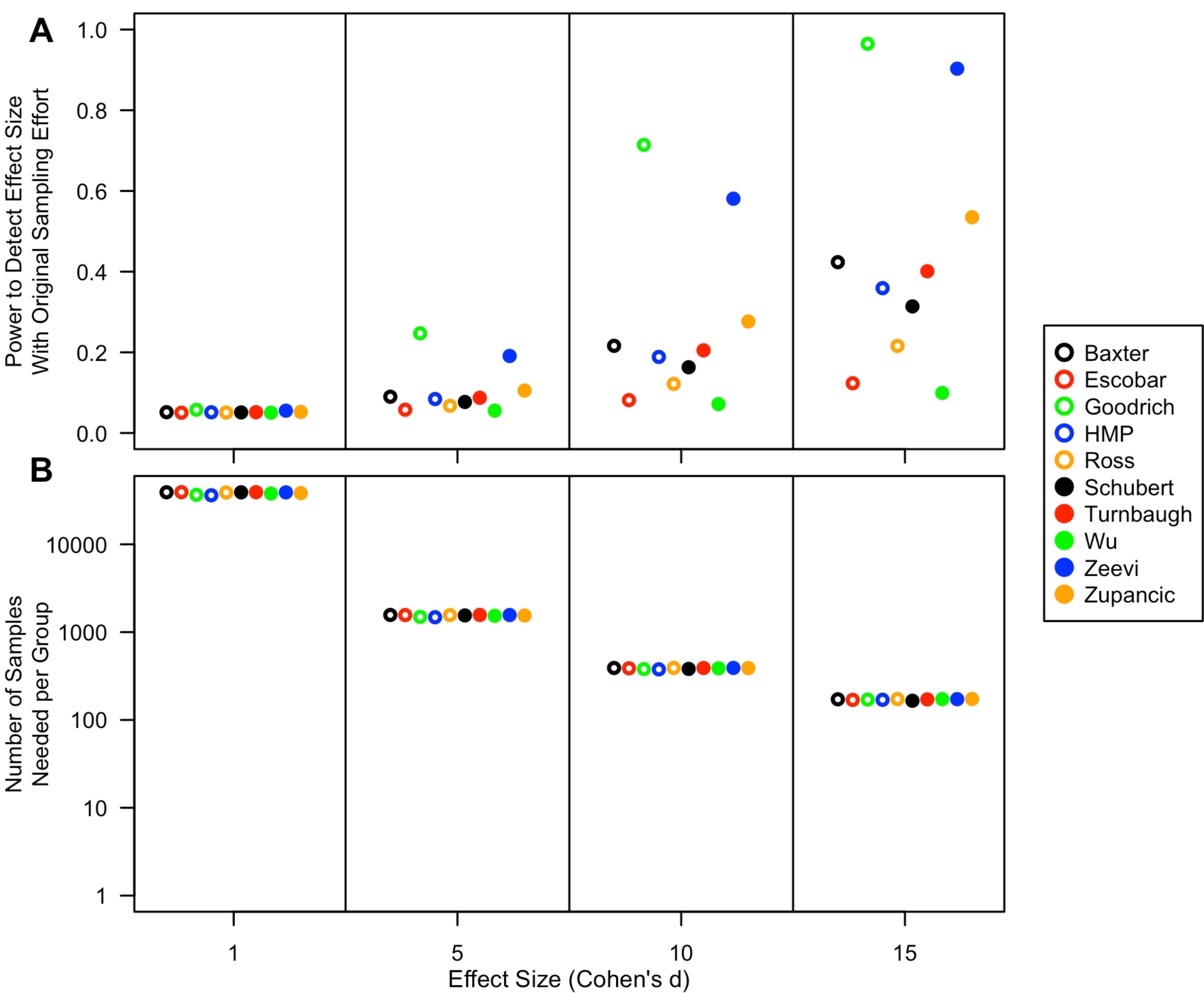

Supplement: Figure S10 — Power (A) and sample size (B) simulations for RR of obesity based on Shannon diversity. Power calculations use the sampling distribution from the original studies, and the sample size estimations assume the same amount of sampling from each treatment group. Download [file mbo004162954sf10.pdf]
